# Spinal morphine but not ziconotide or gabapentin analgesia is affected by alternative splicing of voltage-gated calcium channel Ca_V_2.2 pre-mRNA

**DOI:** 10.1186/1744-8069-9-67

**Published:** 2013-12-26

**Authors:** Yu-Qiu Jiang, Arturo Andrade, Diane Lipscombe

**Affiliations:** 1 Department of Neuroscience, Brown University, Providence, Rhode Island, USA; 2 Department of Physiology, Pharmacology & Neuroscience, City College of the City University of New York, 160 Convent Av, New York, NY 10031, USA

**Keywords:** Voltage-gated calcium channels, Neuropathic pain, Alternative splicing, Morphine, Ziconotide, Gabapentin, Nociception, Analgesia, Spared nerve injury

## Abstract

Presynaptic voltage-gated calcium Ca_V_2.2 channels play a privileged role in spinal level sensitization following peripheral nerve injury. Direct and indirect inhibitors of Ca_V_2.2 channel activity in spinal dorsal horn are analgesic in chronic pain states. Ca_V_2.2 channels represent a family of splice isoforms that are expressed in different combinations according to cell-type. A pair of mutually exclusive exons in the Ca_V_2.2 encoding *Cacna1b* gene, e37a and e37b, differentially influence morphine analgesia. In mice that lack exon e37a, which is enriched in nociceptors, the analgesic efficacy of intrathecal morphine against noxious thermal stimuli is reduced. Here we ask if sequences unique to e37a influence: the development of abnormal thermal and mechanical sensitivity associated with peripheral nerve injury; and the actions of two other classes of analgesics that owe part or all of their efficacy to Ca_V_2.2 channel inhibition. We find that: i) the analgesic efficacy of morphine, but not ziconotide or gabapentin, is reduced in mice lacking e37a, ii) the induction and maintenance of behaviors associated with sensitization that accompany peripheral nerve injury, do not require e37a-specific sequence, iii) intrathecal morphine, but not ziconotide or gabapentin analgesia to thermal stimuli is significantly lower in wild-type mice after peripheral nerve injury, iv) the analgesic efficacy of ziconotide and gabapentin to mechanical stimuli is reduced following nerve injury, and iv) intrathecal morphine analgesia to thermal stimuli in mice lacking e37a is not further reduced by peripheral nerve injury. Our findings show that the analgesic action of morphine, but not ziconotide or gabapentin, to thermal stimuli is linked to which *Cacna1b* exon, e37a or e37b, is selected during alternative pre-mRNA splicing.

## Background

Neuropathic pain can develop following peripheral nerve injury. Symptoms include persistent mechanical hypersensitivity, thermal hypersensitivity, and spontaneous pain [1]. Maladaptive changes in the expression and activity of several ion channels in sensory neurons are implicated in the development and maintenance of certain chronic and neuropathic pain states [2].

**Figure 1:**
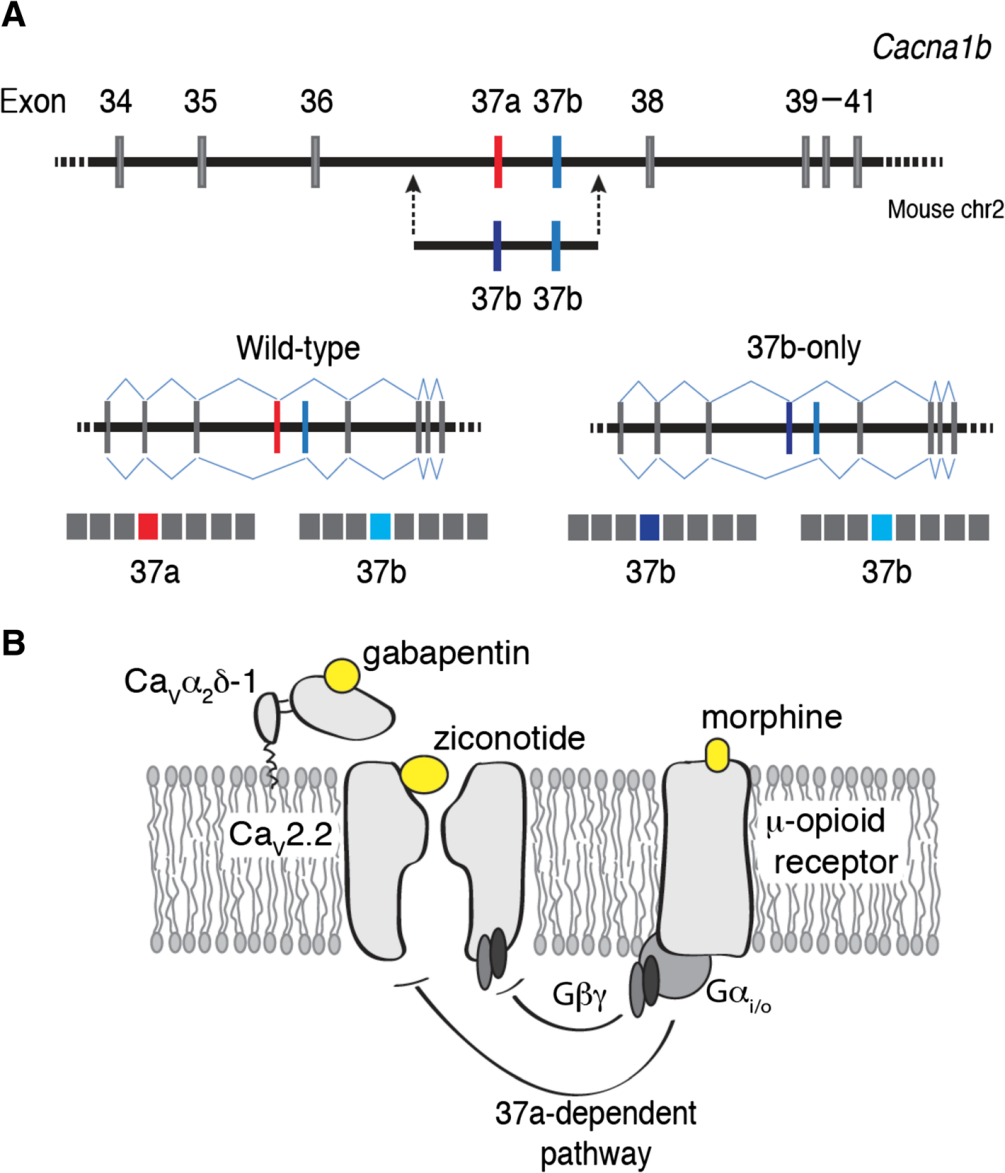
***Cacna1b *exon targeting strategy, and sites of action of three analgesics on Ca**_**V**_**2.2 channels. A,** Illustrates the mouse *Cacna1b* gene on chromosome 2 between exons 34 and 41. Constitutive exons (gray bars), mutually exclusive exons (red and blue bars) and introns (horizontal line) are shown. The region in *Cacna1b* targeted by homologous recombination is delineated by two arrows. The targeting construct contains a second copy of e37b to replace wild-type e37a as previously reported [26]. The replacement e37b (dark blue) contains a silent mutation that introduces a unique *Xho I* site to allow genotyping and tracking of the substituted exon. The possible mRNAs generated from wild-type and e37b-only mice are shown. **B,** illustrates the sites of actions of gabapentin (Ca_V_α_2_δ-1), ziconotide (Ca_V_2.2 α1-subunit) and morphine (μ-opioid receptor). Morphine action on Ca_V_2.2-e37a and Ca_V_2.2-e37b channels is different. Ca_V_2.2-e37a channels are inhibited by both voltage-dependent, Gβγ-dependent and voltage-independent, Gβγ-independent pathways, whereas Ca_V_2.2-e37b channels are mostly sensitive to voltage-dependent, Gβγ-dependent inhibition.

Ca_V_2.2 channels localize to synapses of primary nociceptive neurons in the dorsal horn laminae I/II [3] and they contribute to the control of neurotransmitter release from sensory presynaptic terminals [4,5]. Ca_V_2.2 knockout mice are viable, but have impaired nociception [6-8] and reduced hyperalgesia and mechanical allodynia typically associated with nerve injury [8]. The activity of Ca_V_2.2 channels may be up-regulated following peripheral nerve injury, an event that could contribute to the sensory hypersensitivity that characterizes neuropathic pain [9]. The therapeutic activities of three different classes of analgesics depend fully or partly on their ability to inhibit Ca_V_2.2 channels: Intrathecal ziconotide (ω-conotoxin MVIIA), a selective Ca_V_2.2 blocker, is an effective analgesic in humans with otherwise intractable neuropathic pain including pain from cancer and AIDS [10,11]; opiate analgesics inhibit presynaptic Ca_V_2.2 channels in the spinal dorsal horn via G_i/o_ protein coupled μ-opioid receptors (MORs) [12-17] and gabapentinoids act by binding to the calcium channel Ca_V_α_2_δ-1 subunit to reduce Ca_V_2 channel activity, in part, via effects on protein trafficking [18-20] (Figure 1B).

Ca_V_2.2 channel currents recorded in neurons represent the collective activity of several Ca_V_2.2 channel splice isoforms with their own unique biophysical features [21]. Notably, the exon composition of Ca_V_2.2 splice isoforms is cell-specific and under the control of cell-specific splicing factors [22]. Mutually exclusive exons, e37a and e37b, are present in Ca_V_2.2 mRNAs of nociceptors whereas, e37a-containing Ca_V_2.2 mRNAs are in low abundance elsewhere in the nervous system [23-25]. Levels of e37a-Ca_V_2.2 mRNAs are reduced in DRG 14 days after peripheral nerve injury [24]. Injury-induced decrease in e37a-Ca_V_2.2 mRNA levels likely reduces e37a-specific contributions to synaptic transmission.

Mutually exclusive e37a and e37b encode the proximal 32 amino acids in the C-terminus of Ca_V_2.2 and during pre-mRNA splicing either one or the other is selected [23]. Nociceptors are more likely to express a combination of e37a-Ca_V_2.2 and e37b-Ca_V_2.2 mRNAs compared to other neurons that express primarily e37b-Ca_V_2.2 mRNAs [23,25]. E37a enhances the inhibitory actions of opioids on Ca_V_2.2 channels in cell lines and in neurons, through a G_i/o_ protein-dependent, stimulus-independent mechanism [26,27]. To assess the role of e37a in nociception, we replaced e37a with e37b in the mouse *Cacna1b* gene and showed that e37b-only mice behaved as wild-type in response to noxious thermal stimuli, but intrathecal morphine was a less effective analgesia to thermal stimuli in e37b-only mice compared to wild-type [26].

Here we analyze the role of e37a in: i) the development and maintenance of thermal and mechanical hypersensitivity that accompanies peripheral nerve injury, ii) the analgesic actions of ziconotide and gabapentin, and iii) the analgesic actions of intrathecal morphine, ziconotide, and gabapentin in mice post injury. We conclude that e37a of *Cacna1b* has a specific role, not mimicked by e37b, in spinal morphine analgesia, whereas e37a and e37b are functionally interchangeable in mechanical and thermal nociception.

## Results

**Figure 2:**
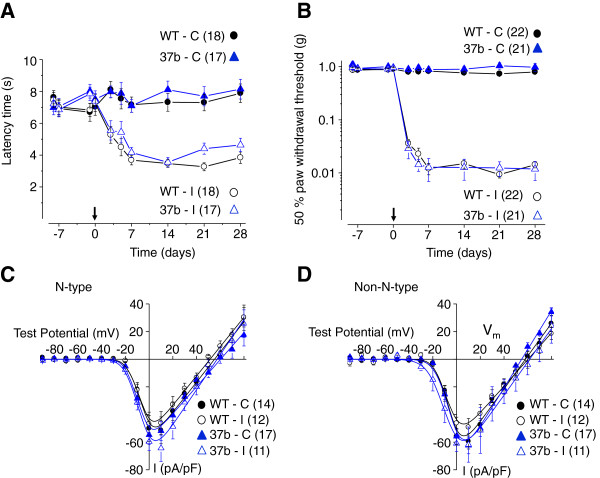
**Development of thermal and mechanical hypersensitivity after peripheral nerve injury in wild-type and e37b-only mice. A**, Paw withdrawal latency to noxious thermal stimuli applied to hindpaws ipsilateral (I) and contralateral (C) to the injury in wild-type (WT) and e37b-only (e37b) mice (Hargreaves method). Latencies were measured at the time points indicated before and after injury (at time 0). *P* values comparing latencies in WT and e37b-only mice were > 0.05 at all time points except at day 21 (*P* = 0.015). At days -8, -7, -1, 0, 3, 5, 7, 14, 21 and 28, *P* values were 0.37, 0.81, 0.07, 0.52, 0.85, 0.74, 0.90, 0.30, 0.71, and 0.87 contralateral; *P* values were 0.72, 0.81, 0.10, 0.87, 0.72, 0.28, 0.29, 0.83, 0.015 and 0.16 for ipsilateral. **B**, Paw withdrawal thresholds to mechanical stimuli applied to hindpaws in WT and e37b-only mice (von Frey filaments). The 50% paw withdrawal threshold from the same side paw compared between WT and e37b-only mice: At days -8, -7, -1, 0, 3, 5, 7, 14, 21 and 28, *P* values were: 0.26, 0.44, 0.29, 0.63, 0.35, 0.28, 0.60, 0.21, 0.05 and 0.18 for contralateral; *P* values were 0.37, 0.72, 0.74, 0.65, 0.47, 0.24, 0.89, 0.67, 0.36 and 0.57 for ipsilateral. Ca_V_2.2 currents measured from capsaicin-responsive neurons of dorsal root ganglia L4, L5 and L6. Total calcium channel currents were recorded and current voltage-relationships generated in the absence and in the presence of ω-conotoxin GVIA **(D)**. Ca_V_2.2 current was generated by toxin-subtraction **(C)**. There is no consistent difference between Ca_V_2.2 current and non-Ca_V_2.2 currents (CgTx-resistant) measured under the different conditions. Only cells responding to 1 μM capsaicin were analyzed. Values are average ± SE. Student’s two-tailed t-test.

In this study, we test the role of e37a in comparison to e37b, to behavioral responses evoked by thermal and mechanical stimuli. We use mice in which we replaced e37a in *Cacna1b* with a second copy of e37b - a manipulation that removes the contribution of e37a without altering total Ca_V_2.2 protein levels (Figure 1A) [26]. E37a-Ca_V_2.2 channels are enriched in TRPV1-containing nociceptors but e37b-only mice respond like wild-type mice to thermal and mechanical stimuli [26]. Therefore, we conclude that e37b substitutes functionally for e37a in basal nociception (see Figure 2; 7 day time point prior to injury).

Levels of e37a-Ca_V_2.2 mRNA decrease in DRG following peripheral nerve injury. We therefore tested if e37a contributes to the development and maintenance of the hypersensitivity that accompanies peripheral nerve injury. Figure 2 shows that mice lacking e37a behave as wild-type in the spared nerve injury (SNI) model of chronic pain. Enhanced sensitivity to noxious thermal and mechanical stimuli is not significantly different in time course or magnitude between wild-type and e37b-only mice. Behavioral responses were maximal in ipsilateral but not contralateral hind paws one week following SNI surgery and were maintained over the 4-week monitoring period. The expression and maintenance of behaviors that accompany SNI in e37b-only mice progressed as in wild-type mice. We conclude that e37a-Ca_V_2.2 and e37b-Ca_V_2.2 channels are equivalent in their capacity to support enhanced thermal and mechanical responsiveness after SNI.

We also measured whole cell voltage-gated calcium (Ca_V_) channel currents in capsaicin-responsive, small diameter neurons of dorsal root ganglia (DRG) L4, L5, and L6 ipsilateral and contralateral to the injured nerve, from WT and e37b-only mice. Endogenous Ca_V_2.2 channel currents measure the overall expression levels of functional Ca_V_2.2 channels on the surface of sensory neurons of wild-type and e37b-only mice. DRG neurons express multiple classes of Ca_V_ channels and the most reliable method to isolate pure Ca_V_2.2 currents is by ω-conotoxin GVIA (CgTx)-sensitivity. Ca_V_ currents were recorded before (total Ca_V_ current) and after 2 μM CgTx to selectively inhibit all Ca_V_2.2 channels and, by toxin-subtraction, Ca_V_2.2 currents were separated from total Ca_V_ current. I-V plots show the average CgTx-sensitive (Ca_V_2.2) and CgTx-insensitive (non-Ca_V_2.2) calcium currents measured from contralateral and ipsilateral neurons of wild-type and e37b-only mice. Ca_V_ current densities in nociceptors are similar across all conditions (Figure 2C, 2D) demonstrating that Ca_V_2.2 channels are expressed at approximately equal levels in DRG of e37b-only and wild-type mice [26]. Furthermore, the basic biophysical features of Ca_V_2.2 currents in nociceptors are not altered by SNI.

**Figure 3:**
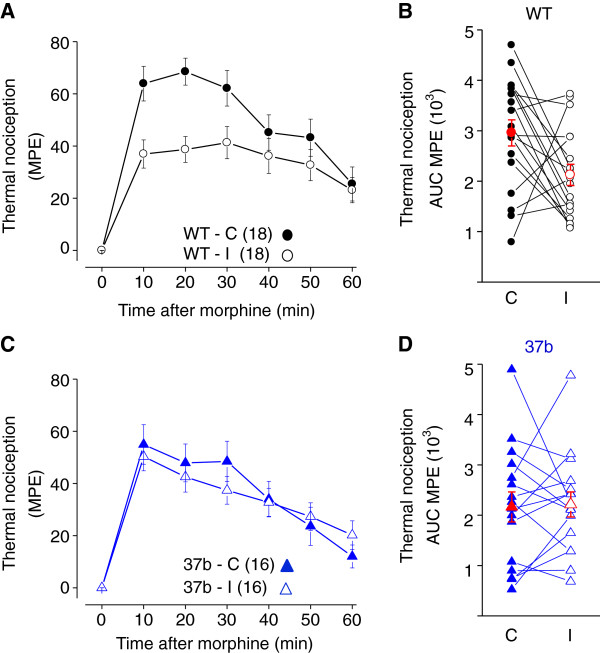
**Percent maximum possible effect of intrathecal morphine after injury in wild-type and e37b-only mice.** Latency to paw withdrawal from thermal stimulus after intrathecal morphine injection (3 μg morphine sulfate) converted into percent maximum possible effect (MPE) using formula: (PWLmorphine - PWLbaseline) × 100/(20 - PWLbaseline) for each time point indicated. **A**, Comparison of spinal morphine effect in response to noxious thermal stimuli in WT mice measured from paws contralateral (WT-C) and ipsilateral (WT-I) to the site of injury. *P* values at 0, 10, 20, 30, 40, 50 and 60 min after morphine injection were 0.32, 0.001, 1.5 × 10^-5^, 0.007, 0.4, 0.42 and 0.91. **B**, Area under the curve (AUC) of the %MPE was calculated for each ipsilateral and contralateral paw and measurements from the same animals connected by a line. There is a significant reduction in AUC for morphine analgesia in ipsilateral compared to contralateral measurements (*P* = 0.0174). **C**, Comparison of spinal morphine effect in response to noxious thermal stimuli in e37b-only mice measured from paws contralateral (37b-C) and ipsilateral (37b-I) to the site of injury. *P* values at 0, 10, 20, 30, 40, 50 and 60 min after morphine injection were 0.13, 0.76, 0.74, 0.44, 0.89, 0.66, 0.36. **D**, Area under the curve of the %MPE was calculated for ipsilateral and contralateral measurements, connecting lines show measurements within each animal. Average values and SE are shown (red symbols). There is no significant difference in AUC for morphine analgesia in ipsilateral compared to contralateral measurements (*P* = 0.777). (Student’s two-tailed t-test for all comparisons).

We showed previously (also see Figure 3A, 3C), that e37a contributes significantly to the spinal level analgesic actions of morphine [26] and that e37a-Ca_V_2.2 mRNA levels are reduced in ipsilateral but not contralateral DRG, following peripheral nerve injury [24]. Here we compare the maximum possible effect (MPE) of intrathecal morphine to lengthen paw withdrawal latencies in response to thermal stimuli, contralateral and ipsilateral to the injured nerve. The %MPE of morphine peaked within 10–20 mins of injection in wild-type mice but morphine efficacy against thermal stimuli applied ipsilateral was substantially lower as compared to contralateral testing (~ 30%, Figure 3A, 3B; *P* = 0.017, paired two-tailed Student’s t-test) [28,29]. Reduced morphine efficacy in peripheral nerve injury animal models may have relevance to morphine tolerance in neuropathic pain states in humans [30]. Interestingly, intrathecal morphine analgesia was similar ipsilateral and contralateral to the injured nerve against noxious thermal stimuli in e37b-only mice (Figure 3A, 3C) (*P* = 0.777 ipsilateral compared to contralateral, paired two-tailed Student’s t-test). We conclude that e37a confers properties, not found in e37b, which contribute to the analgesic actions of morphine. In addition, the presence of e37a-Ca_V_2.2 channels appear to be necessary for the characteristic lowering of morphine analgesia to noxious thermal stimuli that accompanies peripheral nerve injury.

In contrast to its analgesic actions against noxious thermal stimuli, intrathecal morphine was ineffective against noxious mechanical stimuli in both wild-type and e37b-only mice and regardless of nerve injury (data not shown). The lack of intrathecal morphine analgesia against mechanical stimuli is consistent with reports of others [31-33] and with recent findings showing restricted expression of MORs in TRPV1-containing DRG neurons, which respond to noxious thermal stimuli, but not in neurons that respond to noxious mechanical stimuli [34].

Our findings suggest that amino acids encoded by e37a, absent in e37b, are critical for the full inhibitory action of MORs on Ca_V_2.2 channels. We showed previously that e37a enhances the inhibitory actions of opioids on Ca_V_2.2 channels in cell lines and in neurons, through a G_i/o_ protein-dependent, stimulus-independent mechanism [26,27]. However, our experiments do not exclude the possibility that Ca_V_2.2-e37b channels in nociceptor afferents, as compared to Ca_V_2.2-e37a, are simply less sensitive to other drugs that act on Ca_V_2.2 channels independent of G proteins.

Ziconotide (*Conus magnus* peptide ω-conotoxin MVIIA) is a selective peptide inhibitor of Ca_V_2.2 channels and it occludes the central ion pore (Figure 1B) [35,36]. In humans, ziconotide alleviates symptoms of intractable chronic pain but unlike opiates, patients do not develop tolerance during treatment [10,37].

**Figure 4:**
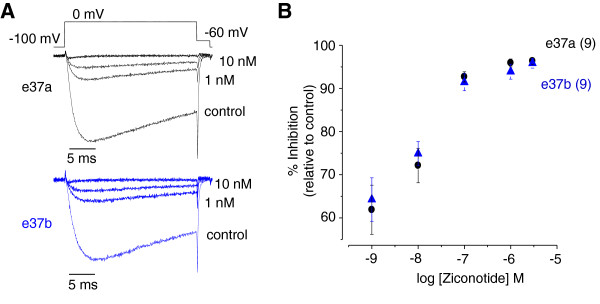
**Ca**_**V**_**2.2 channel splice isoforms inhibited similarly by ziconotide.** Ziconotide inhibits Ca_V_2.2 channel currents in a dose-dependent manner. Ca_V_2.2 cDNAs for each splice isoform were transiently expressed in tsA201 cells together with necessary auxiliary subunits. Calcium channel currents in cells expressing e37a and e37b-Ca_V_2.2 splice isoforms were inhibited by increasing amounts of ziconotide applied to the bath. **A**, representative calcium currents in the presence of different concentrations of ziconotide for each splice isoform (e37a and e37b). Currents were activated by voltage step to 0 mV from a holding potential of -100 mV. **B**, Partial dose–response curve showing the potency of ziconotide to inhibit Ca_V_2.2 calcium currents activated by a voltage-step to 0 mV, from cells expressing either e37a or e37b-Ca_V_2.2 channels.

We compared the inhibitory actions of ziconotide on e37a-Ca_V_2.2 and e37b-Ca_V_2.2 channels transiently expressed in tsA201 cells because its effects on these isoforms have not been reported previously. Ziconotide (1 nM - 3 μM) inhibited e37a-Ca_V_2.2 and e37b-Ca_V_2.2 channel isoforms equally well; ~70% of channels were inhibited at concentrations between 1 nM and 10 µM (Figure 4). We did not test concentrations of ziconotide below 1 nM using whole cell recording because of the long incubation time required to achieve steady-state inhibition.

**Figure 5:**
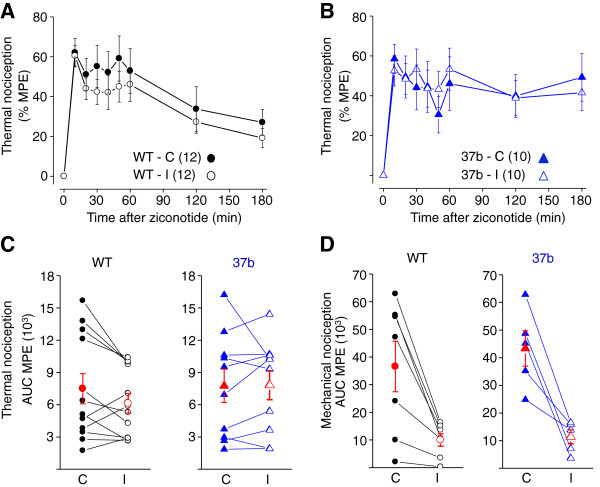
**Analgesic actions of intrathecal ziconotide against noxious thermal and mechanical stimuli in nerve injury model. A**, Latency to paw withdrawal from thermal stimulus after intrathecal ziconotide injection (10 pmol) represented as percent maximum possible effect (MPE) for each time point indicated. **A**, **B**, Comparison of spinal ziconotide analgesia in response to noxious thermal stimuli in WT **(A)** and e37b-only **(B)** mice measured from paws contralateral (C) and ipsilateral (I) to the site of injury. *P* values at all time points were > 0.05. **C**, **D**, Area under the curve of %MPE calculated for each ipsilateral and contralateral paw in response to noxious thermal **(C)** and mechanical **(D)** stimuli for WT and e37b mice plotted individually for each animal and connected by a line. Averages are also shown (red). There is a significant decrease in ziconotide efficacy after injury in WT and e37b-only mice (*P* = 0.017 and 0.002, respectively) whereas there was no difference between genotypes (WT compared to e37b-only *P* = 0.59 for contralateral and 0.69 for ipsilateral measurements). Student’s two-tailed t-test for all comparisons.

We next compared the actions of intrathecal ziconotide (10 pmol, 27 ng) in wild-type and e37b-only mice. Intrathecal ziconotide rapidly prolonged paw withdrawal latencies to both thermal and mechanical stimuli. The MPE of ziconotide peaked within 10 mins (Figure 5A, 5B) and declined slowly over the 3 hr monitoring period. There were no significant differences in the analgesic actions of ziconotide against noxious thermal stimuli applied to hind paws, contralateral and ipsilateral to the site of injury, between wild-type and e37b-only mice (Figure 5C; *P* = 0.42 and *P* = 0.97). Similarly, ziconotide was equally effective as an analgesic in behavioral responses to mechanical stimuli applied to hind paws, contralateral and ispilateral to the injured nerve, in wild-type and e37b-only mice (Figure 5D; *P* = 0.59 and *P* = 0.67). In contrast to intrathecal morphine, ziconotide was effective against noxious mechanical stimuli, but had reduced efficacy against stimuli applied ipsilateral to the injured nerve similarly in wild-type (*P* = 0.016, contralateral compared to ipsilateral) and e37b-only mice and (*P* = 0.002, contralateral compared to ipsilateral) (Figure 5D).

Finally, for completeness we tested the actions of gabapentin in wild-type and e37b-only mice. Gabapentin is an effective treatment in postherpetic neuralgia and other types of neuropathic pain [38]. Although there is little evidence for acute, direct inhibitory actions of gabapentin and its analogue pregabalin on Ca_V_2.2 channels [20,39], their analgesic activity depends on the Ca_V_α_2_δ-1 subunit that binds to, and modulates the trafficking and activity of Ca_V_2.2 channels [40]. We compared the activity of intrathecal gabapentin (100 μg), as previously described above, by measuring paw withdrawal latencies to thermal stimuli and thresholds to mechanical stimuli applied to hind paws ipsilateral and contralateral to the injured nerve. The analgesic effects of intrathecal gabapentin peaked within 10 mins following injection and declined slowly over the 3 hr measurement period.

**Figure 6:**
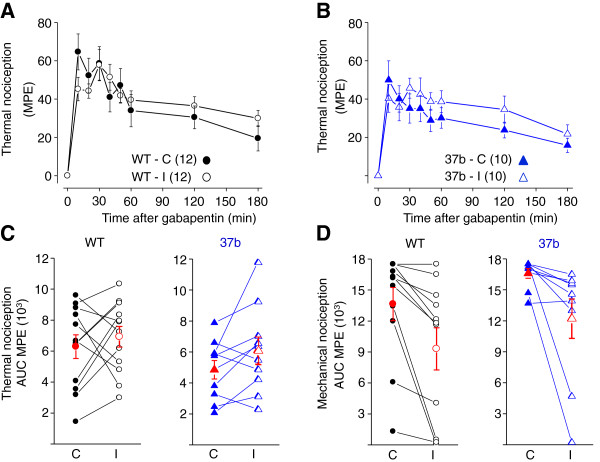
**Analgesic actions of intrathecal gabapentin against noxious thermal and mechanical stimuli.** Latency to paw withdrawal from thermal stimulus after intrathecal gabapentin injection (100 μg) represented as percent maximum possible effect (MPE) for each time point indicated. **A**, **B**, Comparison of spinal gabapentin analgesia in response to noxious thermal stimuli in WT **(A)** and e37b-only **(B)** mice measured from paws contralateral (C) and ipsilateral (I) to the site of injury. *P* values at all time points were > 0.05. **C**, **D**, Area under the curve of %MPE calculated for each ipsilateral and contralateral paw in response to noxious thermal **(C)** and mechanical **(D)** stimuli for WT and e37b mice plotted individually for each animal and connected by a line. Averages are also shown (red). Gabapentin analgesia against noxious mechanical stimuli was slightly but not significantly less effective ipsilateral compared to contralateral to the injured nerve in WT mice (*P* = 0.11) and less effective ipsilateral compared to contralateral to the injured nerve in e37b-only mice (*P* = 0.04). Student’s two-tailed t-test.

The maximum possible effect of gabapentin to thermal and mechanical stimuli applied contralateral and ipsilateral to the injured nerve were not significantly different comparing between wild-type and e37b-only mice (Figure 6; thermal: *P* = 0.17 and *P* = 0.47; mechanical: *P* = 0.12 and *P* = 0.32). Injury did not significantly affect the analgesic actions of gabapentin against noxious thermal stimuli (WT: *P* = 0.54; e37b-only: *P* = 0.26) its actions were reduced against noxious mechanical stimuli in e37b-only mice (*P* = 0.04). Nonetheless, gabapentin was still highly effective as an analgesic after injury (Figure 6D) when compared to ziconotide (Figure 5D).

## Discussion

Ca_V_2.2 channels at afferent terminals in spinal dorsal horn dominate in controlling glutamate and likely substance P release from afferents in lamina I/II of the spinal dorsal horn [5,13,41]. As importantly, Ca_V_2.2 channel are essential for supporting the maladaptive hypersensitivity that accompanies peripheral nerve injury as evidenced by the effectiveness of Ca_V_2.2 channel inhibitors to alleviate behavioral symptoms associated with chronic pain [10,37,42]. It is therefore essential to define the precise class of Ca_V_2.2 channels expressed in nociceptors that are the therapeutic targets of MORs and ziconotide. In this study we support the hypothesis that e37a-Ca_V_2.2 channels are targeted by spinal morphine and that morphine analgesia depends on the presence of e37a-Ca_V_2.2 channels. When e37a-Ca_V_2.2 channels are replaced with e37b-Ca_V_2.2 channels, spinal morphine is less effective against noxious thermal stimuli but importantly, the analgesic actions of ziconotide that inhibits both isoforms equally well are unaffected when e37a is replaced with e37b. However, nerve injury did reduce the analgesic efficacy of ziconotide to mechanical stimuli. Ziconotide and gabapentin, owe all or part of their analgesic efficacy to inhibition of Ca_V_2.2 channels in dorsal horn [10,18,19]. Our findings suggest that cellular factors, that influence cell-specific splicing of Ca_V_2.2 pre-mRNAs, may in turn influence the spinal level actions of morphine.

We do not know the identity of the splicing factors that presumably regulate the selection of e37a over e37b or *vice versa*, during alternative splicing of Ca_V_2.2 pre-mRNA. However, we have shown previously that levels of e37a-Ca_V_2.2 mRNAs decrease selectively in injured but not in uninjured dorsal root ganglia of wild-type mice [24]. Interestingly, the reduction in spinal morphine analgesia to noxious thermal stimuli that accompanies peripheral nerve injury (Figure 3) - precisely when maximal therapeutic efficacy is desirable - is similar in overall magnitude to the reduced spinal morphine analgesia in e37b-only mice compared to wild-type (Figure 3). Moreover, in mice lacking e37a, nerve injury does not further reduce morphine analgesia, an observation that raises the possibility that nerve injury and loss of e37a share a common pathway with respect to morphine action.

In superficial lamina of spinal dorsal horn, MORs inhibit presynaptic Ca_V_2.2 channels as well as activate postsynaptic G protein coupled inwardly rectifying potassium GIRK2 channels [43]. However, presynaptic Ca_V_2.2 channels appear to be the main targets of MOR inhibition of monosynaptic Aδ- and C-fiber-evoked excitatory postsynaptic currents in lamina I neurons and are thus positioned at the critical first junction of the pain transduction pathway [13].

Intrathecal morphine analgesia to noxious thermal stimuli is influenced by the expression of the e37a splice isoform of Ca_V_2.2, whereas ziconotide and gabapentin analgesia are not. We also find that the analgesic efficacies of gabapentin and most strikingly of ziconotide to mechanical stimuli are reduced following nerve injury (Figures 5D, 6D). Our findings raise the possibility that presynaptic channels other than Ca_V_2.2 may dominate in the transmission of noxious mechanical stimuli after nerve injury. Ziconotide and gabapentin are used clinically as analgesics to relieve the symptoms of neuropathic pain [37,44-47] but neither act on Ca_V_2.2 channels via GPCRs or are influenced by e37a [11,39,48,49]. Thus functionally, e37b substitutes for e37a in many cellular activities involving Ca_V_2.2 channels, but not fully in G_i/o_PCR signaling to the channel.

Morphine analgesia to thermal stimuli is influenced by e37a and e37b cannot fully substitute for e37a in mediating its actions on Ca_V_2.2 channels. This is consistent with what we know about the mechanism of G_i/o_PCR inhibition of Ca_V_2.2 channels including by MORs. Whereas all Ca_V_2.2 channel isoforms we have studied to date are inhibited by G_i/o_PCRs via a Gβγ-dependent mechanism that is voltage-dependent, Ca_V_2.2 channels containing e37a are additionally susceptible to inhibition by a mechanism that is Gβγ-independent and voltage-independent (Figure 1B; [26,27]). Similar to our findings, spinal level morphine would therefore be most effective under conditions that favored the presence of e37a-Ca_V_2.2 at afferent terminals and during periods of high neuronal activity (Figure 1A) [26,27].

Although in this study we have focused on Ca_V_2.2 splice isoforms as modulators of the anti-nociceptive actions of morphine, other receptors and ion channels, most notably MORs, are also critical. For example, MOR levels are significantly reduced in DRG neurons following nerve injury [50]. Thus, the direction of changes in both MOR and its target Ca_V_2.2-e37a channel in DRG following injury combine to reduce morphine action in spinal dorsal horn. By contrast, ziconotide analgesia against noxious thermal stimuli is independent of nerve injury, Ca_V_2.2 channel splice isoform, and MOR expression levels. In the clinic, ziconotide use is limited by side effects that include psychoses, and these are not generally tolerated except by the most severely ill patients [10,11,37,42]. Thus, small molecule inhibitors of presynaptic Ca_V_2.2 channels with preferential action in the spinal dorsal horn could be therapeutically valuable. In this regard, drugs designed to preferentially target and inhibit e37a-specific sequences or e37a-specific Ca_V_2.2 channel activity could be analgesic and have reduced side effects (because of reduced action on other Ca_V_2.2 splice isoforms). Even though e37a-Ca_V_2.2 mRNA levels fall in DRG following nerve injury, acute knockdown of e37a-Ca_V_2.2 mRNAs by siRNA reverses thermal hypersensitivity that accompanies nerve injury [24]. Thus it is possible that e37a-Ca_V_2.2 channels are preferentially expressed at nociceptor terminals in spinal dorsal horn and as such represent a valuable therapeutic target to alleviate neuropathic pain [24,26].

Substantial evidence points to extensive alternative splicing of multi-exon mammalian genes according to cell-type, developmental stage, disease, and injury [21]. It can be technically challenging to determine the cellular and behavioral consequences of individual alternative splicing events when isoform-specific drugs and antibodies are lacking. Nonetheless, our exon-replacement strategy demonstrates the functional significance of an individual alternatively spliced exon in the *Cacna1b* gene in the context of thermal nociception. The more precisely we can define cell-specific isoforms of major ion channel and receptor drug targets, within their appropriate cellular and disease contexts, the more precisely we can inform therapeutic strategies.

## Methods

### Animals

Animal housing and experimental procedures were carried in accordance with the Brown Institutional Animal Care and Use Committee guidelines. 2–3 month old male mice of mixed genetic background were used (predominantly 129 and C57BL6). Wild-type mice were bred in parallel with e37b-only mice to obtain matched genetic backgrounds [26]. The e37b-only mouse line contains a second copy of e37b, replacing e37a, in the *Cacna1b* gene [26]. Nociceptors of dorsal root ganglia from wild-type mice express both e37a and e37b isoforms [23].

### Surgery

Spared nerve injury model (SNI) was performed according to methods described in [51]. Briefly, after exposing the three branches of the sciatic nerve (common peroneal, tibial and sural) in anesthetized mice, the common peroneal and tibial branches were carefully segregated from surrounding tissues, tightly ligated and axotomized ~2 mm of the distal nerve stump. The sural branch was left intact. Behavioral analyses were performed on days 7 and 1 before surgery, on the day of surgery (0) and then 1, 3, 5, 7, 14, 21 and 28 days after surgery.

### Behavioral analyses

We measured sensitivity of the hindpaw to thermal and mechanical stimuli in wild-type and e37b-only mice contralateral and ipsilateral to the injury. To assess thermal sensitivity, we measured paw withdrawal latencies using Plantar Testing Instruments (IITC) [52]. A high-intensity beam (setting = 20%, ~45 W) was applied to the middle of the plantar surface of a resting mouse. Paw-withdrawal latency (PWL) was measured to the nearest 0.1 s with a cut-off value of 20 s. To assess mechanical sensitivities we measured thresholds for paw withdrawal, using up and down method, in response to a graded series of von Frey filaments (0.008, 0.02, 0.04, 0.07, 0.16, 0.4, 1, 2 g) applied to the glabrous surface of the hind paw. The maximum mechanical stimulus applied was 2 g [53]. To determine analgesia efficacies, we injected 3 μg morphine sulfate, 10 pmol ziconotide or 100 μg gabapentin intrathecally using a 28 gauge needle connected to a micro syringe through a 15 PE 50 polyester catheter (15 cm). Mice were anesthetized with 2% isofluorane during the entire surgery [44]. We monitored the analgesic effects of each drug measuring paw withdrawal latencies to a thermal stimulus and paw withdraw thresholds to mechanical stimuli before and at 10 min intervals following drug injection, testing continued for 60 mins for morphine and 3 hrs for ziconotide and gabapentin. Analgesia efficacy was calculated as % maximum possible effect for wild-type and e37a-lacking mice: %MPE = (PWL_drug_ - PWL_baseline_) *100/(20 - PWL_baseline_). The area under the curve of % MPE (AUC) was used to compare overall drug efficacies. Each measurement reflects average data from at least 10 different animals for each condition.

### Electrophysiology on acutely dissociated dorsal root ganglia neurons

Whole cell patch clamp recordings were obtained from individual neurons acutely dissociated from DRG L4, L5 and L6 ipsilateral and contralateral to the site of injury. DRG were isolated 14–28 days after surgery and behavioral testing was performed to confirm the presence of hypersensitivity to thermal and mechanical stimuli. Voltage-gated calcium currents were recorded as described previously using 1 mM calcium as the charge carrier [26]. All neurons were challenged with 1 μM capsaicin and only capsaicin-responsive neurons were included in Figure 2. The fraction of capsaicin-responsive and non-responsive neurons was the same independent of injury and genotype.

### Ziconotide inhibition of Ca_V_2.2 splice isoforms tested in tsA201 cells

We used tsA201 cells to transiently express Ca_V_2.2 e37a and e37b splice isoforms Genbank AF055477 & AF055477. All Lipscombe clones used in our study are deposited with Addgene. Cells were transfected using lipofectamine 2000 (Invitrogen) with one of either e37a-Ca_V_2.2 or e37b-Ca_V_2.2 cDNA together with Ca_V_β_3_ (Lipscombe/Addgene plasmid 26574) Ca_V_α_2_δ-1 (Lipscombe/Addgene Plasmid 26575) and eGFP (Clontech). After 24 hours of expression, we performed standard whole cell patch clamp recordings as described in [27]. Extracellular solution (mM): 1 CaCl_2_, 4 MgCl_2_, 10 HEPES, 135 tetraethyl ammonium chloride (TEA-Cl), pH 7.2 with TEAOH. Internal solution (mM): 126 CsCl, 10 EGTA, 1 EDTA, 10 HEPES, 4 MgATP, pH 7.2 with CsOH. Ziconotide was perfused onto the cell using fine glass pipettes placed in close proximity as used in [26].

### Statistical analysis

In all experiments including behavioral analyses and electrophysiology analyses the experimenter collecting data was blinded to genotype and clone. The genotype and clone identities were revealed only after all data were collected and analyzed. Data presented are mean ± SE. Comparison of mean differences between wild-type and e37b-only mice were made by unpaired two-tailed Student’s *t*-test. Comparison of mean differences between contralateral and ipsilateral threshold values within individual genotypes were made by paired two-tailed Student’s *t*-test. Where relevant, *P* values are shown in text.

## Abbreviations

MPE: Maximum possible effect; SNI: Spared nerve injury; DRG: Dorsal root ganglia.

## Competing interests

Authors declare that they have no competing interests.

## Authors’ contributions

Y-QJ carried out surgeries and behavioral studies, AA carried out electrophysiology studies. AA and DL wrote the manuscript. All authors performed analyses. All authors read and approved this manuscript.
